# Adapting the HIV Infant Tracking System to Support Prevention of Mother-to-Child Transmission of HIV in Kenya: Protocol for an Intervention Development Pilot Study in Two Hospitals

**DOI:** 10.2196/13268

**Published:** 2019-06-08

**Authors:** Sarah Finocchario-Kessler, May Maloba, Melinda Brown, Brad Gautney, Kathy Goggin, Catherine Wexler, Natabhona Mabachi, Beryne Odeny, Silas Lagat, Sharon Koech, Jacinda K Dariotis, Thomas A Odeny

**Affiliations:** 1 Department of Family Medicine University of Kansas Medical Center Kansas City, KS United States; 2 Global Health Innovations Nairobi Kenya; 3 Global Health Innovations Dallas, TX United States; 4 School of Medicine University of Missouri-Kansas City Kansas City, MO United States; 5 Health Services and Outcomes Research Children’s Mercy Kansas City Kansas City, MO United States; 6 School of Pharmacy University of Missouri-Kansas City Kansas City, MO United States; 7 Ministry of Health Nandi County Kapsabet Kenya; 8 Center for Microbiology Research Kenya Medical Research Institute Nairobi Kenya; 9 College of Education, Criminal Justice & Human Services University of Cincinnati Cincinnati, OH United States

**Keywords:** HIV, eHealth, mHealth, pregnancy, retention, medication adherence, infant, diagnosis, Kenya

## Abstract

**Background:**

Despite progress to expand access to HIV testing and treatment during pregnancy in Kenya, gaps still remain in prevention of mother-to-child transmission of HIV (PMTCT) services. This study addresses the need for effective and scalable interventions to support women throughout the continuum of care for PMTCT services in low-resource settings. Our research team has successfully implemented the HIV Infant Tracking System (HITSystem), a Web-based, system-level intervention to improve early infant diagnosis (EID) outcomes.

**Objective:**

This study will expand the scope of the HITSystem to address PMTCT services to bridge the gap between maternal and pediatric HIV services and improve outcomes. This paper describes the intervention development protocol to adapt and pilot an HITSystem version 2.0 to assess acceptability, feasibility, and preliminary PMTCT outcomes in Kenya.

**Methods:**

This is a 3-year intervention development study to adapt the current HITSystem intervention to support a range of PMTCT outcomes including appointment attendance, antiretroviral therapy (ART) adherence, hospital deliveries, and integration of maternal and pediatric HIV services in low-resource settings. The study will be conducted in 3 phases. *Phase 1* will elicit feedback from intervention users (patients and providers) to guide development and refinement of the new PMTCT components and inform optimal implementation. In *Phase 2*, we will design and develop the HITSystem 2.0 features to support key PMTCT outcomes guided by clinical content experts and findings from Phase 1. *Phase 3* will assess complete PMTCT retention (before, during, and after delivery) using a matched randomized pilot study design in 2 hospitals over 18 months. A total of N=108 HIV-positive pregnant women (n=54 per site) will be enrolled and followed from their first PMTCT appointment until infant HIV DNA Polymerase Chain Reaction testing at the target age of 6 weeks (<7 weeks) postnatal.

**Results:**

Funding for this study was received in August 2015, enrollment in Phase 1 began in March 2016, and completion of data collection is expected by May 2019.

**Conclusions:**

This protocol will extend, adapt, and pilot an HITSystem 2.0 version to improve attendance of PMTCT appointments, increase ART adherence and hospital-based deliveries, and prompt EID by 6 weeks postnatal. The HITSystem 2.0 aims to improve the integration of maternal and pediatric HIV services.

**Trial Registration:**

ClinicalTrials.gov NCT02726607; https://clinicaltrials.gov/ct2/show/NCT02726607 (Archived by WebCite at http://www.webcitation.org/78VraLrOb)

**International Registered Report Identifier (IRRID):**

DERR1-10.2196/13268

## Introduction

### Background

Despite impressive progress to expand access to HIV testing and treatment during pregnancy in Kenya [1,2], gaps still remain in achieving comprehensive prevention of mother-to-child transmission of HIV (PMTCT) services. Despite widespread access (77% to 80%) to the highly efficacious Option B+ antiretroviral therapy (ART), the current rate of perinatal transmission in Kenya is estimated at 8.3% to 11.5% [3-5]. Late and inconsistent attendance at antenatal appointments, suboptimal ART adherence during pregnancy and breastfeeding, high rates of unskilled deliveries, and high dropout throughout the PMTCT cascade are among the barriers to eliminating perinatal HIV infection in Kenya [6].

Although Kenyan national guidelines encourage 4 or more antenatal care (ANC) visits [7], an estimated 11.2% to 57.6% [8,9] attend all 4 appointments, with 95% of women attending at least 1 ANC appointment [2]. Attendance of all PMTCT appointments is crucial for monitoring treatment, progression of pregnancy, and managing risk of transmission. Poor medication adherence during pregnancy and breastfeeding compromises the efficacy of ART to prevent HIV transmission. In Kenya, approximately 15% to 20% of pregnant and postpartum women are less than 95% adherent to their ART regimen [10,11]. Since 2012, hospital deliveries are free of charge; yet, nationwide, only 61% of births are facility-based [9], with HIV positive women more likely to have a nonfacility delivery than HIV uninfected women [12]. Home deliveries among HIV positive women miss opportunities for timely infant ART prophylaxis, which can reduce the risk of perinatal transmission by up to 47% in low-resource settings [13-16]. Infants of mothers with suboptimal drug adherence are more vulnerable to perinatal HIV transmission [17,18], thus infant ART prophylaxis remains a critical safeguard.

The PMTCT cascade necessitates that women successfully navigate a series of steps including maternal HIV counseling and testing, initiation and monitoring of ART, delivery care, infant ART and cotrimoxazole prophylaxis, linkage into early infant diagnosis (EID) care for their HIV-exposed infants, and linkage to lifelong HIV care for themselves. Although HIV testing during ANC is high (93.1%) [2], rates of retention after successful linkage to PMTCT are low. In a study conducted by Ayuo et al [19], 31.9% of pregnant women enrolled in PMTCT were disengaged from care for more than 30 consecutive days (thus missing a medication refill appointment) at least once during their pregnancy, with 22.5% of all patients disengaged during the critical phase immediately before delivery. These data reflect a setting with an active outreach program for retention and thus may underestimate attrition in facilities without outreach programs. After delivery, an additional 19.3% to 45% of infants are lost to follow-up [20,21]. At least one quarter of infants are enrolled late in EID, missing the target for EID testing by 6 weeks of age [22]. Consequently, a significant proportion of mother-infant pairs receive incomplete services [23].

Mobile phone and Web-based technologies provide viable solutions to individual and system-level challenges in the delivery of HIV services in Kenya and other African countries**.** Short message service (SMS) text messaging interventions have improved attendance at ANC appointments [24,25], medication adherence and viral suppression [15,16], and postpartum PMTCT retention [26]. Tailoring health messages to the individuals within the target population increases participant retention and is critical to the success of mHealth interventions [27-29]. Most mHealth interventions for EID have focused on lab-specific efficiency [30], patient-specific adherence, or retention support [31], whereas the HIV Infant Tracking System (HITSystem) mHealth intervention was the first to link laboratory, clinical, and patient stakeholders in one integrated system [32].

This study addresses the need for effective and scalable interventions to coordinate the continuum of care for PMTCT services in low-resource settings to maximize retention and minimize infant HIV infection. Our research team has successfully implemented a Web-based mHealth intervention called the HITSystem, which utilizes available technology (internet and texting) to improve communication and accountability between EID stakeholders to optimize outcomes for HIV-exposed infants [33,34]. The HITSystem is unique as a *system-level* mHealth intervention combining SMS outreach to mothers of HIV-exposed infants and algorithm-based dashboard alerts to prompt provider (maternal and child health [MCH]/HIV and laboratory) action, and it demonstrated significant reductions in turnaround times for Polymerase Chain Reaction (PCR) test results (2.3 weeks faster), mother notification of results (1.3 weeks faster), younger infant age at ART initiation (7.6 weeks younger), and higher retention throughout the complete 18-month EID cascade of care (85% vs 60%; aOR 3.7 (2.5 to 5.5); *P*<.001) when compared with standard EID services [32]. This proposal responds to requests from hospital administrators and health care providers to expand the scope of the HITSystem to include antenatal PMTCT services and to bridge the current gap between maternal and pediatric HIV services. Thus, this intervention development protocol will adapt and pilot an HITSystem version 2.0 that prospectively tracks and supports HIV positive women through their pregnancy and delivery, linking them to EID follow-up at birth (initial HITSystem 1.0) to facilitate linked mother-infant data and retention. We will assess the acceptability, feasibility, and preliminary impact on PMTCT retention of the HITSystem 2.0 intervention.

### Study Overview

**Figure 1:**
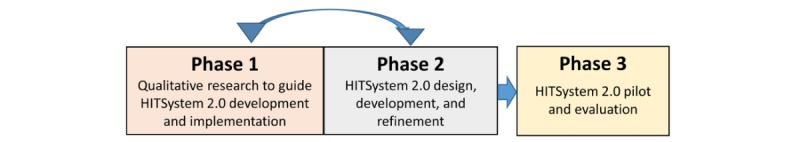
Study phases—an iterative process of qualitative research and HIV Infant Tracking System (HITSystem) 2.0 design, development, and refinement, which precedes the HITSystem 2.0 pilot and evaluation.

This is a 3-year intervention development study to adapt and pilot test an extension of the current HITSystem intervention to support a range of PMTCT outcomes including retention in ANC, ART adherence, hospital deliveries, and integration of maternal and pediatric HIV services in low-resource settings. The study will be conducted in 3 phases (Figure 1). *Phase 1* is designed to elicit feedback from intervention users (patients and providers) to guide development of the new PMTCT components and inform optimal implementation at the intervention site. *Phase 2* involves the technical design and development of HITSystem 2.0, a new iteration of the HITSystem to support key PMTCT outcomes (appointment attendance, ART medication adherence, hospital-based delivery, and linkage to EID). Phase 2 development will be guided by clinical content experts and findings from Phase 1. This involves close collaboration with programmers and system users to refine the interface and customize options. *Phase 3* will pilot the HITSystem 2.0 in 1 hospital over an 18-month period and compare targeted PMTCT outcomes with those at a matched control hospital.

## Methods

### Phase 1: Formative Research

Phase 1 seeks to understand optimal HITSystem SMS text messaging strategies. We draw on the Information Processing Communication Theory [35-37], which identifies the conditions that encourage people to actively attend to health messages, to guide questions regarding text message content and timing. We will recruit HIV positive pregnant and postpartum women who are/were enrolled in PMTCT care at the intervention site to participate in 3 focus groups to elicit (1) strategic communication and customization of SMS text messages to motivate target behaviors while protecting confidentiality, (2) barriers to patient retention and ART adherence, and (3) strategies for mobile phone outreach and alternatives for patient follow-up. Focus groups will include n=8 women, with 2 of the 3 focus groups including HIV-positive pregnant women (second and third trimester) and the third including HIV-positive postpartum women (2 to 16 weeks), thus representing a range of gestational and postpartum periods. In addition, key informant interviews will be conducted with all staff engaged in PMTCT services (antenatal, maternity, postnatal, laboratory, pharmacy, and mentor mothers; approximately n=8) at the intervention site to strategize optimal HITSystem 2.0 implementation, given PMTCT provider capacity and existing systems for patient flow. In addition to notetaking, with participants’ consent, the focus groups and interviews will be digitally recorded. Participants will receive 500 Kenyan Shillings (approximately US $5) in appreciation of their time. The research protocol was reviewed and approved by IRB at the Kenya Medical Research Institute in Nairobi, Kenya, and the University of Kansas Medical Center. Informed consent was conducted with all participants before study engagement.

#### Analyses for Focus Group and Key Informant Interview Data

Audio files will be translated and transcribed in English, coded, and analyzed. In total, 2 study team members will independently code data for a priori (preferences for health messaging, potential barriers to retention and ART adherence, alternative strategies for patient follow-up, and provider recommendations for optimal implementation) and emergent themes. Through consensus, we will develop a codebook with typical exemplars for each theme, noting the frequency and distribution of themes within the larger topic areas. Summarized themes will directly inform HITSystem 2.0 development in Phase 2.

### Phase 2: Intervention Development

HITSystem 1.0 is currently in use in multiple health facilities in Kenya. The primary goals of the HITSystem 1.0 are to (1) improve EID and clinical management of HIV-exposed infants and (2) facilitate early ART initiation for HIV positive infants [33,34,38,39]. The HITSystem is accessed on the Web using a computer or tablet, with mobile broadband modems that respond to a cellular signal. The primary components include (1) provider alerts to complete time-sensitive interventions, (2) real-time communication of PCR laboratory results to hospitals to reduce turnaround time, (3) persistent follow-up for timely ART initiation among HIV-positive infants, and (4) retention in EID care via SMS text messaging and/or patient tracing.

The HITSystem 2.0 intervention will (1) utilize electronic prompts to notify PMTCT providers and program managers when action is required to support timely services, patient retention and adherence, and linkage to EID once infants are born [33,34] and (2) send SMS text messages to HIV-positive pregnant women’s mobile phones with the aim to motivate adherence to medication, remind women of ANC appointments and medication refills, and prompt preparation for a hospital delivery.

#### HIV Infant Tracking System 2.0 Intervention Specifications

Clear reporting of intervention components and implementation strategies is needed for meaningful application in practice or testing in research [40,41]. The entry point into the HITSystem 2.0 will be the first PMTCT appointment for women previously diagnosed with HIV or newly confirmed HIV positive during antenatal HIV screening. Actions include automated prompts to PMTCT providers, prompts to central laboratory technicians who process viral load (VL) samples, and SMS texts to patients driven by national algorithms for optimal PMTCT care (Table 1). Automated electronic prompts to providers will persist as Web-based dashboard alerts until addressed. Automated SMS texts are sent to HIV-positive pregnant women as cues to action; women can select the preferred content and frequency for one-way ART adherence text messages at the time of enrollment. This is one of the first interventions to target adherence support messages during pregnancy, thus texting options (content and frequency) will be informed by Phase 1 formative research, and actual preferences will be assessed by this pilot study. Community health care workers can reach out to patients who miss services and/or do not respond to SMS text messages or calls to encourage retention. The HITSystem 2.0 will integrate PMTCT and EID services through 1 coordinated system-level intervention; this will permit longitudinal follow-up of mother-infant pairs to assess PMTCT outcomes.

**Table 1:** HITSystem 2.0 intervention components. Expected timing, dose, and target of alerts and SMS (short message service) texts to providers and HIV positive pregnant women.

Actors	Actions	Timing	Dose^a^	Target
Hospital-based providers; providers	Algorithm-driven electronic alerts	When PMTCT^b^ service late/missing (enrollment-infant EID^c^ link)	Avg=8 per mom, range=0 to 14+	PMTCT quality: complete PMTCT retention (pregnancy to EID link)
Lab-based providers; lab techs	Algorithm-driven electronic alerts	When receipt of VL^d^ sample or VL results delayed	Avg=1 per mom, range=0-2+	PMTCT efficiency: reduced turn-around time for key PMTCT services
HIV-positive pregnant women	Algorithm-driven electronic SMS text messages	Appointment reminders (2 days before appointment); adherence reminders (daily, weekly, biweekly, monthly); facility-based delivery reminders (4 and 2 weeks before EDD^e^)	Avg=4 per mom, range=0-8+; avg=20 per mom, range=0-245+; avg=2 per mom, range=2 to 2	PMTCT quality and PMTCT efficiency

^a^Dose represents anticipated study averages and ranges only, assuming one alert per alert type per pregnancy. Actual dose will depend on client-specific factors such as gestational age at enrollment, adherence to PMTCT guidelines and scheduled services, and frequency preferences for adherence reminders.

^b^PMTCT: prevention of mother-to-child transmission of HIV.

^c^EID: early infant diagnosis.

^d^VL: viral load.

^e^EDD: estimated due date.

#### Process for Phase 2 Development

We will employ the 4D’s process to define, design, develop, and deploy the intervention [42]. The clinical algorithms and portions of the system development will be designed before completion of Phase 1, but focus group and interview findings will customize and refine critical aspects of the 4D’s process, incorporating input from Kenya’s current PMTCT treatment guidelines and user feedback regarding strategic messaging, patient and provider barriers, and strategic implementation. The iterative process of Phases 1 and 2 will enhance the design and development by ensuring a user-friendly, robust, interactive, and secure software system to increase engagement, retention, and quality PMTCT outcomes.

### Phase 3: Trial Intervention

#### Trial Design

We will employ a matched, randomized pilot study design in 2 government hospitals with comparable resources, patient volume, and basic patient characteristics. One hospital will be randomized to receive the HITSystem 2.0 intervention and the other hospital will maintain standard of care PMTCT services. An extended 18-month implementation period will allow 9 months to reach the sample size requirement of n=54 at each hospital and another 9 months to ensure complete prospective outcome data from the first PMTCT appointment in pregnancy until the infant’s first HIV DNA PCR test result is determined. This follow-up period will assess complete retention throughout PMTCT services, ART adherence, and rates of infant HIV transmission. We will also collect retrospective PMTCT outcome data from each hospital for the 18 months before implementation to facilitate pre- and postcomparisons at the same facility.

Satisfaction interviews with HITSystem 2.0 users (HIV positive women and providers) will inform barriers to use that can be targeted either through future strategic refinement of the design or collaboration with key partners to address constraints beyond the scope of this intervention.

#### Study Sites and Setting

This study will be conducted in 2 government hospitals with similar provider-patient ratios and staffing structure, Kapsabet Hospital and Bungoma Hospital, both in western Kenya. Eligibility criteria for these hospitals include government funding; the current provision of PMTCT, EID, and ART services; at least one dedicated provider managing PMTCT services; and maintenance of individual-level patient files. Kapsabet Hospital serves a catchment area of approximately 91,000 people [43], has an average monthly EID volume of 10, and an average infant HIV transmission rate of 2.7% [44]. Bungoma Hospital serves a catchment area of approximately 92,412 people [43], has an average monthly EID volume of 18, and an average infant HIV transmission rate of 4.2% [44]. These hospitals serve less transient local populations, making them ideal pilot sites to adapt and modify the HITSystem 2.0 for the primary outcome of complete PMTCT retention.

#### Randomization

As a system-level intervention, it is neither feasible nor acceptable to randomize participation at the individual level. The hospital to receive the intervention will be determined by random assignment at the beginning of the study, using a random number generator program. The principal investigator and research staff will be blind to the process of assigning the sites.

#### Participant Eligibility

All HIV-positive pregnant women presenting for their first PMTCT appointment at the intervention and control site will be eligible for enrollment in the study. Women at the intervention site must also own or have reliable access to a mobile phone.

#### Procedures

Participants at both sites will receive PMTCT services guided by the Kenyan national guidelines [7]. At the time of PMTCT enrollment (first visit), all eligible women will be informed about the study by the clinical staff, and written informed consent for participation, including a review of hospital records documenting PMTCT services, will be obtained by a trained hospital staff member.

##### Training of Hospital and Study Staff

Key hospital personnel (PMTCT and maternity nurses and mentor mothers) will be trained by employing hands-on data entry scenarios tailored to the specific capacity in which they will utilize the HITSystem 2.0. During the first weeks of implementation, the trained research assistant (RA) will work closely to ensure thorough understanding and accurate independent use of the HITSystem 2.0 and will be available throughout the study to retrain or assist in system-related problem solving. The RA will be based in the MCH department with PMTCT providers to monitor and support fidelity to intervention implementation and timely data entry into the HITSystem 2.0. The US-based HITSystem team will remotely review the entered data to identify any challenges or systematic errors to be addressed. This is the same training and implementation process successfully employed for the HITSystem pilot [33] and cluster randomized trial to evaluate EID outcomes [32]. Training at the control site will be limited to assessing participant eligibility, conducting informed consent, and administering the baseline survey. Nurses and mentor mothers directly involved in PMTCT service provision will conduct these research tasks. All research and clinical staff involved in the study will be trained on procedures to ensure participant confidentiality and secure data storage.

*Baseline surveys* will be conducted at the initial PMTCT enrollment visit at both intervention and control sites for all eligible women. In addition to the basic demographic information captured in routine care at both hospitals (age and number of children), questions will assess (1) knowledge and perceived importance of attendance at scheduled appointments; ART before, during, and after delivery; consequences of poor adherence; and infant testing, (2) motivation and self-efficacy to complete the targeted behaviors (eg, attendance, medication adherence, plan for delivery, and prompt EID enrollment) using scaled and Likert responses, (3) assessment of depression symptoms (6 questions to assess frequency of symptoms between 0 to 7 days a week) and risk for intimate partner violence (3 screening questions to facilitate counseling referral), and (4) male partner involvement. Study staff will enter completed paper surveys in Excel 2016 (version 1708).

*PMTCT service utilization* will be documented in the patient file and relevant PMTCT-related facility registers (intervention and control) as required by the Kenyan Ministry of Health. At the intervention hospital, a new record will be created in the HITSystem 2.0 during PMTCT enrollment and will be updated at each subsequent visit, including mobile phone number(s) and residence tracing information. Prospective clinical data from the mother-infant pair will be captured in the HITSystem 2.0 to track medication adherence, appointment attendance, delivery outcomes, and postpartum infant prophylaxis data, automating provider alerts and SMS text messages to participants as indicated. Any information that is not captured in the HITSystem at the time of the patient’s visit (owing to workflow, connectivity, or other challenges) will be updated as soon as possible after the visit, referencing any relevant paper-based patient and facility records needed. All relevant data to measure the targeted PMTCT outcomes will be entered and maintained in the HITSystem 2.0.

At the control site, paper-based patient files and facility registries are the primary record-keeping source for PMTCT data. A study staff member will visit the control site quarterly to review clinical records to document and update PMTCT services received by women enrolled in the study. Multiple sources will be cross-referenced to collect standard of care data at the control hospital: (1) pregnant women/mothers’ medical records, (2) hospital ANC registry book, (3) pharmacy ART registry, (4) maternity registry, and (5) HIV-exposed infant registry.

*HITSystem 2.0 user satisfaction* will be assessed with semistructured interviews to solicit feedback on usefulness and acceptability of the content and frequency of the targeted SMS text messages, patient tracing efforts, and other recommendations to improve the intervention. Participants (n=35) will be purposively selected to include variation among women with complete/incomplete retention, low/high ART adherence, and facility/home deliveries. Interviews will be conducted in the language they prefer (Swahili or English) once study participation is complete. We will also conduct interviews with providers and hospital administrators to better understand the strengths and limitations of the HITSystem 2.0 implementation, its integration with the current HITSystem 1.0*,* and suggestions for improvement. Remuneration for interview participation and the analysis plan for qualitative data will be the same as described in Phase 1. Interviews will not be conducted at the control site as they did not experience the intervention.

##### Fidelity Assurance Procedures

Standard operating procedures were developed for each step of the study process to standardize training, enrollment, and all data collection procedures. As it is Web-based, any modification or corrections to the HITSystem are instantly applied across all users. HITSystem entries are routinely reviewed to ensure completeness and appropriate utilization of the comments section. Study personnel will make quarterly visits to the control site to cross-reference enrollment records with patient files and the relevant PMTCT-related facility registers and to review informed consent documents and surveys for accuracy, completeness, and secure storage.

### Primary Outcome: Complete Prevention of Mother-to-Child Transmission of HIV Retention

The primary outcome is complete PMTCT retention, measured from the first PMTCT appointment until the infant’s first HIV test, which is targeted at 6 weeks of age. The aggregate measure of complete retention—calculated as retention before delivery, at delivery, and after delivery—will be dichotomously coded as complete or incomplete retention. Complete retention is defined as maternal ART initiated (if not already on ART at the time of pregnancy), all scheduled PMTCT appointments attended, hospital-based delivery, and HIV DNA PCR testing by 6 weeks (<7 weeks) postnatal.

### Secondary Outcomes

#### Duration of Prevention of Mother-to-Child Transmission of HIV Retention

We will estimate the duration (number of weeks) of PMTCT retention for each participant by subtracting the last date of documented service engagement from the date of enrollment. This will allow comparisons between total time retained in PMTCT services between groups and identify when (antenatal, delivery, or early postnatal) disengagement from PMTCT most often occurs.

#### Prevention of Mother-to-Child Transmission of HIV Appointment Attendance

We will measure the proportion of mothers who attended all scheduled ANC appointments, documenting late (>7 days past scheduled date) and missing (>14 days past scheduled date) attendance. The number of possible appointments will vary on gestational age at PMTCT enrollment; thus we will measure the median number of visits, the proportion who received the recommended 4 or more appointments, and the proportion enrolled by the recommended 14 weeks gestation.

#### Viral Load Suppression

We will document the proportion of pregnant women who had a VL sample collected during pregnancy: 6 months after ART initiation for those diagnosed during ANC or within the past 6 months for those already established on ART, in accordance with national guidelines. We will abstract data on VL from the HITSystem 2.0 (intervention) and the patient file and laboratory records (control) to assess the proportion with VL tests taken, the turn-around time (TAT) for return of results, and the proportion with suppressed VL results (defined as<1000 viral copies/ml) during pregnancy.

#### Medication Adherence

As VL is still not consistently collected during pregnancy, we will rely on other indicators of adherence including self-report of missed doses in the 7 days before appointment (prescribed vs taken) and pill counts conducted by relevant providers (dispensed vs returned). Adherence data will be entered in the HITSystem 2.0 at each appointment. We will calculate the proportion of patients who report >95% adherence to doses prescribed (7-day self-report) and pill count.

*Hospital delivery* will be estimated from the proportion of pregnant mothers who deliver at a health facility. Women who do not deliver at the study hospital will be contacted within 2 weeks of their estimated due date to check on their progress, document location of birth, and encourage prompt return to the facility for postpartum and postnatal care. *EID enrollment* will be measured using the proportion of HIV-exposed infants tested by 6 weeks of age (<7 weeks).

*Infant and maternal mortality* will be reported separately, noting all available data on date and cause of death. A future study will utilize a design with a longer follow-up period and larger sample size to adequately evaluate perinatal transmission of HIV.

#### Power and Sample Size

The required sample size was calculated to compare differences in proportions of complete PMTCT retention for the intervention (p1) and control (p2). We estimated complete retention by combining available retention data from Kenya for each phase: antenatal retention (68%) [19], hospital-based deliveries (61%) [36], and postnatal retention for HIV-exposed infants within 12 weeks (54.9% [21] to 80.7% [20]; average 67%). Recognizing that these estimates do not represent cumulative risk for the same individuals, we conservatively estimate complete PMTCT at the control site to be 20%. Our EID pilot data showed that retention doubled at 9 months with retention rates as high as 94.2% [33]. Using a midrange between differences achieved from published studies and our own pilot study, we conservatively estimate complete retention of 47% for HITSystem 2.0 participants. A sample size of 108 (54 intervention and 54 control participants) is needed to achieve 80% power to detect a significant difference in the proportion of complete PMTCT retention with chi-square tests (goodness of fit test: contingency table) with an effect size of 0.27 and a 2-tailed alpha of .05. Accounting for the proportion with mobile phone utilization, and 1.5% maternal mortality [45] and 3.7% [46] perinatal mortality, we estimate n=70 eligible participants. During Phase 3 enrollment, we plan to enroll n=54 of the n=70 eligible PMTCT patients per hospital. On the basis of our initial HITSystem pilot where <1% of women declined, we anticipate high participation.

#### Statistical Analyses

To examine the baseline participant and facility characteristics, we will calculate descriptive statistics including proportions for categorical variables and means (SD) or median (interquartile range) for continuous variables [47]. For the primary outcome, chi-square tests will be used to compare the proportion with complete PMTCT retention between groups [48]. We will explore correlations between complete PMTCT retention and possible predictors (eg, knowledge, motivation to complete care, maternal HIV disclosure, perceived benefit of ART, maternal depression, and risk of intimate partner violence) using Phi, point-biserial, or Spearman rho statistics depending on the measurement level of each predictor [49]. Analytic models will control for significantly different preintervention covariates across conditions and hospitals, clustered observations (eg, time nested within setting), and gestational age as it impacts the eligible/recommended number of appointments or services.

For secondary analyses, Kaplan-Meier curves will illustrate differences in the duration of PMTCT retention before, during, and after delivery, accounting for the woman’s eligible period of engagement (based on the timing of enrollment and accounting for premature infant or maternal mortality). We will then identify time periods when meaningful decreases in PMTCT retention occur, using Cox proportional-hazard regression for survival analyses (ie, retention), if all assumptions are met [50]. Known variables associated with HIV service retention (age, disclosure status, educational level, and number of children [22,51,52]) and those correlated (*P*<.10) will then be included in a generalized linear mixed model to identify a parsimonious list of predictors.

Using chi-square tests, we will compare the proportion of pregnant women (1) attending all scheduled PMTCT appointments (4 or more PMTCT visits and late or missed visits), (2) tested for VL (VL sample collected, results returned, and detectible VL values), (3) delivery at the hospital, (4) enrolling in EID (dried blood spot sample collected by <7 weeks), and (5) have an infant diagnosed with HIV, between intervention and control sites. The median number of PMTCT appointments, median gestational week at first PMTCT appointment, and median TAT for VL results will be calculated and compared using Mann Whitney *U* tests. Although medication adherence documentation at the control site (typically described as poor, fair, or good) will not permit direct comparison, the proportion of pregnant women at the intervention site with >95% adherence will be described. Participant preferences for the content and frequency of SMS text messages for medication adherence support will also be described. *P* values will be adjusted (using Tukey correction) for multiple tests, where appropriate. Quantitative data will be analyzed using Statistical Application Software (SAS) version 9.4.

## Results

We are actively enrolling participants and collecting data at the intervention and control sites. Only formative data have been analyzed and the HITSystem has been adapted. There are no planned interim analyses of the primary outcome. No analyses comparing intervention versus control outcome data have been conducted at this time.

## Discussion

Previous HITSystem pilot data and studies have demonstrated significant success in improvement of EID outcomes particularly in improving the efficiency and quality of key EID outcomes [32-34,38]. Leveraging the improved linkage of health facilities, mother-infant pairs, and laboratories in the context of EID, the HITSystem 2.0 (PMTCT) will expand the current Web-based platform to bridge the gap between maternal and pediatric HIV services. Outcomes at each step of the PMTCT cascade will be analyzed, and specific challenges will be identified.

This study will design and implement the HITSystem 2.0 through formative qualitative research, intervention development using the 4Ds process, and a pilot implementation period as described above. It will also provide user satisfaction data by highlighting the HITSystem 2.0’s usefulness, acceptability, challenges, and recommendations to improve the intervention. The proposed HITSystem 2.0 will integrate maternal and infant HIV services in one system-level, Web-based intervention that will provide linked, prospective data for mother-infant pairs, which has been identified as a problematic gap in the current system. The findings of this study will refine the HITSystem 2.0 intervention, particularly tracking and utilization of challenging measures such as medication adherence and antenatal VL results to enhance system performance and implementation strategies in a larger scale efficacy trial.

Although this intervention will focus on health system improvement to enhance quality of care for women and infants in the facility setting, the study will not directly target PMTCT uptake, which would require community-based outreach. Given that intervention site data will be tracked via the HITSystem whereas control site data will be captured via disparate paper-based registries and patient files, there may be discrepancies in data quality and documentation of services rendered between control and intervention sites; all efforts will be made to capture any available data at control sites to minimize this limitation. As the study design and implementation evolve, we anticipate that eHealth technologies in the study regions will likewise evolve and influence the standard of PMTCT care. These external factors will be documented and accounted for in the design and analyses.

## Appendix

Multimedia Appendix 1 Peer-reviewer report from the National Institutes of Health.

## Abbreviations

ANC: antenatal care
ART: antiretroviral therapy
EDD: estimated due date
EID: early infant diagnosis
HITSystem: HIV Infant Tracking System
MCH: maternal and child health
PCR: Polymerase Chain Reaction
PMTCT: prevention of mother-to-child transmission of HIV
RA: research assistant
SMS: short message service
TAT: turn-around time
VL: viral load
