# Supplementary material for: Adapting the HIV Infant Tracking System to Support Prevention of Mother-to-Child Transmission of HIV in Kenya: Protocol for an Intervention Development Pilot Study in Two Hospitals
Source: JMIR Res Protoc. 2019 Jun 8;8(6):e13268. doi: 10.2196/13268 (PMC6592400; doi:10.2196/13268)
Supplement: Multimedia Appendix 1 [file resprot_v8i6e13268_app1.pdf]

SUMMARY STATEMENT
(Privileged Communication)

PROGRAM CONTACT:
Susannah Allison Ph.D.
240-627-3861
allisonsu@mail.nih.gov

Release Date: **04/09/2015**

Application Number: **1 R34 MH107337-01A1**

Principal Investigator

KESSLER, SARAH FINOCCHARIO PHD

Applicant Organization: UNIVERSITY OF KANSAS MEDICAL CENTER

Review Group: **BSCH**
Behavioral and Social Consequences of HIV/AIDS Study Section
AIDS

Meeting Date: **03/09/2015**
Council: **MAY 2015**
Requested Start: **07/01/2015**

RFA/PA: **PA14-179**
PCC: **9A-ASPA**
Dual IC(s): **AI**

Project Title: **Adapting the HITSystem to Support PMTCT Retention and ART Adherence**

SRG Action: **Impact Score: 21 Percentile: 9 +**
Next Steps: **Visit http://grants.nih.gov/grants/next_steps.htm**
Human Subjects: 30-Human subjects involved - Certified, no SRG concerns
Animal Subjects: 10-No live vertebrate animals involved for competing appl.
Gender: **1A-Both genders, scientifically acceptable**
Minority: **5A-Only foreign subjects, scientifically acceptable**
Children: **1A-Both Children and Adults, scientifically acceptable**
Clinical Research - not NIH-defined Phase III Trial

Project Year	Direct Costs Requested	Estimated Total Cost
1	150,000	211,873
2	150,000	211,873
3	150,000	211,873
TOTAL	450,000	635,619

ADMINISTRATIVE BUDGET NOTE: The budget shown is the requested budget and has not been adjusted to reflect any recommendations made by reviewers. If an award is planned, the costs will be calculated by Institute grants management staff based on the recommendations outlined below in the COMMITTEE BUDGET RECOMMENDATIONS section.

1R34MH107337-01A1 KESSLER, SARAH

RESUME AND SUMMARY OF DISCUSSION: This application proposes to adapt the web-based HIV Infant Tracking System (HITSystem) for pregnant HIV+ women in Kenya in an effort to improve the engagement and retention of these women in reproductive health services before, during, and after delivery. The HITSystem has been used to track HIV-exposed infants and it has improved infant outcomes by providing care alerts to the HIV care providers and by prompting the mothers through SMS text messages to bring in the infant for follow up care. The goal of the intervention is to prevent mother to child transmission (PMTCT) and the development of effective methods for PMTCT is a significant undertaking that can have a broad public health impact. The applicant and her investigative team have the expertise to carry out the proposed study. The committee found this to be an innovative approach to PMTCT and the proposed study is well designed. The applicant has been responsive to concerns raised in the prior review of the application. Concerns about how infant mortality and women not delivering in the clinic will be handled in the data analyses have been addressed, sample size concerns were addressed, and concerns about the preliminary data were clarified. There was a remaining concern about a lack of description of how the team was going to address barriers to care, but this did not diminish the committee's overall high enthusiasm for the many strengths of this responsive resubmission.

DESCRIPTION (provided by applicant): Despite impressive progress to expand access to HIV testing in antenatal care (ANC) and antiretroviral (ARV) prophylaxis to HIV+ pregnant women, Kenya continues to struggle to provide comprehensive HIV prevention and treatment services for women and infants. Innovative interventions that integrate HIV services and target both system and individual level barriers will maximize the impact of existing efforts to eliminate MTCT of HIV globally. Prospective follow-up and support for mother-infant pairs from prevention of mother-to-child transmission (PMTCT) through early infant diagnosis (EID) services can reduce HIV transmission and maximize the quality of maternal and pediatric care. The HIV Infant Tracking System (HITSystem) is a web-based mHealth, system-level intervention designed and evaluated by our research team that has dramatically improved early infant diagnosis (EID) outcomes by providing efficient prospective tracking of HIV-exposed infants, automated electronic action 'alerts' for both providers and lab technicians to prompt 8 time-sensitive EID interventions, and SMS text messages to mothers when return hospital visits are required. Based on the promising impact of the HITSystem in Kenya, we propose to adapt the design and scope of the HITSystem to target PMTCT for HIV+ pregnant women. Our objective in this application is to modify the HITSystem to engage and retain HIV+ pregnant women throughout antenatal, obstetric, and postnatal services and evaluate its impact on PMTCT related behaviors. The research will be conducted in 3 phases. In Phase 1 we will conduct qualitative research with HIV+ pregnant and postpartum women to tailor text message content and timing, and with healthcare providers engaged in PMTCT care to inform the implementation strategy for Phase 3. In Phase 2, we will adapt the programming of algorithm-driven electronic alerts and patient follow-up features to support PMTCT and identify high-risk mothers for additional support. In Phase 3, we will evaluate the HITSystem 2.0 by randomly assigning HITSystem implementation to one of two closely matched government hospitals. The primary outcome is complete PMTCT retention across three phases of care (before, during and after delivery). Secondary outcomes include ART adherence and infant HIV status.

PUBLIC HEALTH RELEVANCE: The potential public health impacts of the proposed research include: 1) improved attendance of prevention of mother-to-child transmission (PMTCT) appointments; 2) improved adherence to HIV antiretroviral regimen during and after pregnancy; 3) increased proportion of deliveries in health care facilities where intrapartum and immediate postpartum prophylaxis can be provided; 4) earlier (at delivery) enrollment of HIV-exposed infants into EID care; and 5) improved integration of maternal and pediatric HIV services.

CRITIQUE 1:

Significance: 2
Investigator(s): 2
Innovation: 2
Approach: 3
Environment: 2

Overall Impact: Current PMTCT strategies in Kenya result in a 15% infection rate among infants, far exceeding the target rate of < 5%. Widespread passive internet-based patient tracking data platforms could be useful for being part of an integrated system to increase access and retention to existing PMTCT strategies for mothers and infants. This project seeks to modify an existing HIV Infant Tracking System (HITSystem) to improve tracking of HIV+ pregnant women in care through to the postpartum period. The project is supported by an experienced team, especially in the area of database and technology development who developed and support the original HITSystem 1.0. This project, if successful, is likely to increase access and retention into PMTCT strategies but to further reduce MTCT rates systems and client factors may need to be considered.

1. Significance:

Strengths

- Addresses the problem of high MTCT rates by increasing access and adherence to EID and PMTCT strategies using the existing widespread HITSystem.
- Capitalizing on widespread use of mobile technologies to facilitate access and adherence to PMTCT services.

Weaknesses

- Technological features of the system are covered but some key drivers of success may be neglected and have the potential to compromise the impact of this technological advancement.

2. Investigator(s):

Strengths

- Multidisciplinary team with expertise in a variety of disciplines including information technology, communications, & HIV care.
- The team is strong in database and technology development as evidenced by their experience and development of the HITSystem 1.0

Weaknesses

- While the team has ample experience with technology development, it is not clear that there is ample expertise in considering the system, workforce, or patient factors that may facilitate or act as barriers to the tracking activities.

3. Innovation:

Strengths

- The expansion of the HITSystem to include tracking around PMTCT services is innovative.

Weaknesses

- Use of mobile technologies is a strength but not an innovation.

4. Approach:

Strengths

- This application addresses the high rate of PMTCT in Kenya which is well above the target rate of $<$ 5%.
- This project builds upon a widespread HIV Infant Tracking System (HITSystem) that is to be expanded to improve access and adherence to PMTCT strategies from the antenatal period through to postpartum.
- The strong mixed methods design of conducting qualitative research to inform the content and timing of the text messages and implementation from the perspective of providers.
- Expertise to modify and design clinical and programmatic algorithms to optimize tracking from the antenatal period thru postpartum.
- Implementation of an evaluation to evaluate outcomes as well as the acceptability and integration with the existing EID focused system.

Weaknesses

- Preliminary data about preferred messages may not reflect the heterogeneity of the target population.
- Phase 1 seems to be focused on messages for those who have few barriers to accessing services with too little attention to that population who might have significant barriers.
- The team clearly has the expertise to modify the HITSystem and related technology. However, to fully increase access and retention in PMTCT strategies, the team should also be considering the system, workforce, or patient factors that may facilitate or act as barriers to the tracking activities. For example, if the system is successful in significantly increasing adherence, how will current capacity be impacted? While the system will generate text messages to motivate target behaviors, what are the potential harms that might be generated and what protections will be put in place to minimize harm (e.g., partner support or exposure to violence if confidential information is disclosed)?

5. Environment:

Strengths

- The environment is appropriate for the proposed research

Weaknesses

- None noted.

Protections for Human Subjects:

Acceptable Risks and/or Adequate Protections

Data and Safety Monitoring Plan (Applicable for Clinical Trials Only):

Acceptable

Inclusion of Women, Minorities and Children and not IRB Exemption #4.

- Sex/Gender: Distribution justified scientifically

- Race/Ethnicity: Distribution justified scientifically
- Inclusion/Exclusion of Children under 21: Including ages < 21 justified scientifically

Vertebrate Animals:

Not Applicable (No Vertebrate Animals)

Biohazards:

Not Applicable (No Biohazards)

Resubmission:

- The applicants were responsive to the comments made by the previous reviewers. The applicants now randomize the matched sites for the evaluation; addressed sample size concerns; addressed concerns about the mortality outcomes; expanded upon their description of the tracking of women not delivering at the hospital; and expanded upon descriptions of prior experience including lessons learned.
- However, while the investigators claim to have focused on `cultural and structural forces that impede care seeking and protective behaviors `this was not readily apparent in the revised application. This is a concern given that their outcomes are adherence to PMTCT-related behaviors and infant HIV status.

Budget and Period of Support:

Recommend as Requested

CRITIQUE 2:

Significance: 1
Investigator(s): 1
Innovation: 2
Approach: 2
Environment: 1

Overall Impact: This R34 application has been very responsive to reviewers and is highly significant in its scope. The proposed modification and upgrade of a mHealth intervention by the research team aims to engage and retain HIV-positive pregnant women through the postpartum period to reduce the incidence of vertical transmission in Kenya. This study builds on previous successful work and represents an ongoing collaboration among the investigators. If successful, this mHealth intervention could be scaled-up but could also be modified for other health outcomes.

1. Significance:

Strengths

- Despite vast progress in expanding access to HIV testing and treatment during pregnancy in Kenya, 15% of HIV-exposed infants still become infected each year.

- The research team successfully implemented a web-based mHealth intervention, HIV Infant Tracking System (HITSystem), which has greatly improved early infant diagnosis outcomes.
- Through text messaging, the proposed modified mHealth intervention, HITSystem 2.0, will remind pregnant women to keep antenatal appointments and will inform providers of missed appointments; it will also send targeted messaging to participants for birth preparedness.
- The modified mHealth intervention will support medication adherence of participants, as well as at the system level to support provider actions.
- The proposed mHealth Intervention will also monitor and support mother-infant pairs throughout the continuum of care.

Weaknesses

- None noted.

2. Investigator(s):

Strengths

- The study team is excellent and has a shared history of conducting successful research.

Weaknesses

- None.

3. Innovation:

Strengths

- The HITSystem 2.0 will provide continuity of care and follow-up for mother-infant pairs across vertical programs.
- HITSystem 2.0 will target the nearly 40% of HIV-exposed infants who are never enrolled in early infant diagnosis (EID) interventions. Tracking strategies will provide novel data on the proportion of mothers seeking postnatal care at local clinics vs. the hospital and informing strategies to increase EID uptake.
- The HITSystem software and communication strategies can be adapted for other health priorities within and beyond HIV care.
- Following women from pregnancy through postpartum will be a unique contribution to the literature.

Weaknesses

- Text messaging is not novel.

4. Approach:

Strengths

- The application has strong theoretical underpinnings and is process-oriented.
- The proposed study builds on strong existing international partnerships.
- A randomized matched control is a strength.
- Although participants will stop being actively followed after 12 weeks postnatal, women will be followed through the prior HITSystem until 18 months postnatal (and data will be tracked).

Weaknesses

- No major weaknesses.

5. Environment:

Strengths

- The research institutions have strong supportive environments.

Weaknesses

- None.

Protections for Human Subjects:

Acceptable Risks and/or Adequate Protections

Data and Safety Monitoring Plan (Applicable for Clinical Trials Only):

Acceptable

Inclusion of Women, Minorities and Children and not IRB Exemption #4.

- Sex/Gender: Distribution justified scientifically
- Race/Ethnicity: Distribution justified scientifically
- Inclusion/Exclusion of Children under 21: Including ages < 21 justified scientifically

Vertebrate Animals:

Not Applicable (No Vertebrate Animals)

Biohazards:

Not Applicable (No Biohazards)

Resubmission:

- Very responsive

Budget and Period of Support:

Recommend as Requested

CRITIQUE 3:

Significance: 2
Investigator(s): 2
Innovation: 2
Approach: 2
Environment: 2

Overall Impact: This is the second submission of an R34 application from an experienced investigator. The project focuses on the HITSystem 2.0, a web-based mhealth health system intervention. Past research addressed mother-infant pairs (HIV-infected mothers) and the present study seeks to apply this model to HIV+ pregnant women to improve retention to PMTCT services. A system-wide mhealth intervention to improve retention in prevention of MTCT services and reduce transmission to infants would have high public health utility. HITSystem is algorithm-based and automated, making it potentially scalable. Further, the study is innovative. The Approach is strong and appropriate for the research questions the application seeks to address. The revised application was responsive to the previous review.

1. Significance:

Strengths

- The project focuses on the HITSystem 2.0, a web-based mhealth health system intervention. Past research addressed mother-infant pairs (HIV-infected mothers) and the present study seeks to apply this model to HIV+ pregnant women to improve retention to PMTCT services. A system-wide mhealth intervention to improve retention in prevention of MTCT services and reduce transmission to infants would have high public health utility.
- 13,000 or 15% of Kenyan infants become infected with HIV each year, pointing the high potential public health significance of the present study.
- Kenya will establish (or is interested in establishing) the HITSystem as a national EID (early infant diagnosis) program. Adding a PMTCT component to the system would have high public health utility.
- HITSystem is algorithm-based and automated, making it potentially scalable.
- HITSytem is compatible with other electronic medical record systems.

Weaknesses

- None noted.

2. Investigator(s):

Strengths

- Dr. Kessler is a relatively new but productive HIV researcher.
- Dr. Kessler was a senior member of the team that developed the HITSystem.
- The team has past experience in health communications research in Kenya.
- Global Health Innovations coordinates the implementation of the HITSystem in Kenya, including coordination with the Ministry of Health.
- The research team in the US and Kenya is strong and has collaborated together in the past.

Weaknesses

- As noted in the previous review, Dr. Finocchario-Kessler is described as a Co-Investigator in her bio sketch (pg 24), but she is the PI.

3. Innovation:

Strengths

- The HITS 2.0 approach, a system-wide mhealth intervention, is innovative.

Weaknesses

- None noted.

4. Approach:

Strengths

- The proposal is grounded in a past study of HITSystem 1.0.
- Pilot data on HITSystem 1.0 are promising, showing increases in EID retention and the proportion of infants on ART in urban and peri-urban areas.
- HITSystem 2.0 will integrate maternal and pediatric services.
- HITSystem 2.0 will target the 40% of HIV-exposed infants who are never enrolled in EID.
- The study is grounded in Information Processing Communication Theory and the IMB model.
- HITSystem 2.0 will be modified to target 8 primary PMTCT behaviors, such as enrollment, pre-treatment lab work, through hospital based delivery, enrollment in EID at delivery, and receipt of the infant's HIV test result. These 8 behaviors appear to capture the range of relevant behaviors as women move from pregnancy to delivery.
- The primary endpoints (PMTCT retention across three phases of care) and secondary endpoints (ART adherence and infant HIV status) appear appropriate for the present study.
- HITSystem 2.0 is designed to address system-level barriers while engaging and motivating patients.
- The intervention will be developed, tested, and then refined.
- 43% of births occur in a health facility and the proposal argues obstacles can be overcome with preparedness planning. HITSystem 2.0 will include birth preparedness messages.
- 85% of Kenyans have mobile phones supporting the feasibility of the text message component of the intervention
- Text messages will be individually tailored to improve their efficacy and improve engagement
- Mothers with less than 85% adherence will be flagged for increased outreach.
- The revised application was responsive to the previous review.

Weaknesses

- The application notes that text messages will be sent to motivate adherence to ART. Motivating adherence to ART is typically a challenge. It is not clear how well the messages can motivate adherence, despite the attention paid to message development in the proposal.
- The facilities and resource section notes that one site has been designated as an intervention site (Kapsabet) and another as a matched control site. The larger proposal notes that sites will be randomly assigned.

5. Environment:

Strengths

- The environments are strong and well positioned to support the present study.

Weaknesses

- None noted

Protections for Human Subjects:

Acceptable Risks and/or Adequate Protections

- pg 53 notes that this is not a clinical trial but it would appear to be a clinical trial. NIH recently released guidance on what constitutes a clinical trial (NOT-OD-15-015)..

Data and Safety Monitoring Plan (Applicable for Clinical Trials Only):

Acceptable

Inclusion of Women, Minorities and Children and not IRB Exemption #4.

- Sex/Gender: Distribution justified scientifically
- Race/Ethnicity: Distribution justified scientifically
- Inclusion/Exclusion of Children under 21: Including ages < 21 justified scientifically

Vertebrate Animals:

Not Applicable (No Vertebrate Animals)

Biohazards:

Not Applicable (No Biohazards)

Budget and Period of Support:

Recommend as Requested

THE FOLLOWING SECTIONS WERE PREPARED BY THE SCIENTIFIC REVIEW OFFICER TO SUMMARIZE THE OUTCOME OF DISCUSSIONS OF THE REVIEW COMMITTEE, OR REVIEWER'S WRITTEN CRITIQUES, ON THE FOLLOWING ISSUES:

PROTECTION OF HUMAN SUBJECTS (Resume): ACCEPTABLE

INCLUSION OF WOMEN PLAN (Resume): ACCEPTABLE

INCLUSION OF MINORITIES PLAN (Resume): ACCEPTABLE

INCLUSION OF CHILDREN PLAN (Resume): ACCEPTABLE

COMMITTEE BUDGET RECOMMENDATIONS: The budget was recommended as requested.

+ Derived from the range of percentile values calculated for the study section that reviewed this application.

NIH has modified its policy regarding the receipt of resubmissions (amended applications). See Guide Notice NOT-OD-14-074 at http://grants.nih.gov/grants/guide/notice-files/NOT-OD-14-074.html. The impact/priority

score is calculated after discussion of an application by averaging the overall scores (1-9) given by all voting reviewers on the committee and multiplying by 10. The criterion scores are submitted prior to the meeting by the individual reviewers assigned to an application, and are not discussed specifically at the review meeting or calculated into the overall impact score. Some applications also receive a percentile ranking. For details on the review process, see http://grants.nih.gov/grants/peer_review_process.htm#scoring.

MEETING ROSTER

Behavioral and Social Consequences of HIV/AIDS Study Section
AIDS and Related Research Integrated Review Group
CENTER FOR SCIENTIFIC REVIEW
BSCH
March 09, 2015 - March 10, 2015

CHAIRPERSON

SEAL, DAVID W, PHD
PROFESSOR
DEPARTMENT OF GLOBAL COMMUNITY HEALTH
AND BEHAVIORAL SCIENCES
SCHOOL OF PUBLIC HEALTH AND TROPICAL MEDICINE
TULANE UNIVERSITY
NEW ORLEANS, LA 70112

MEMBERS

BENOIT, ELLEN , PHD *
INVESTIGATOR
NATIONAL DEVELOPMENT AND
RESEARCH INSTITUTES, INCORPORATED
NEW YORK, NY 10010

BLANK, MICHAEL B, PHD *
ASSOCIATE PROFESSOR
CENTER FOR MENTAL HEALTH POLICY
AND SERVICES RESEARCH
DEPARTMENT OF PSYCHIATRY
UNIVERSITY OF PENNSYLVANIA
PHILADELPHIA, PA 19104

BOGART, LAURA M, PHD
ASSOCIATE PROFESSOR
DEPARTMENT OF MEDICINE
HARVARD MEDICAL SCHOOL
BOSTON, MA 02215

BRADY, STEPHEN MICHAEL, PHD *
ASSOCIATE PROFESSOR
MENTAL HEALTH COUNSELING AND
BEHAVIORAL MEDICINE PROGRAM
DEPARTMENT OF PSYCHIATRY
BOSTON UNIVERSITY SCHOOL OF MEDICINE
BOSTON, MA 02118

BROUWER, KIMBERLY C, PHD *
ASSOCIATE PROFESSOR
DEPARTMENT OF MEDICINE
DIVISION OF GLOBAL HEALTH
UNIVERSITY OF CALIFORNIA, SAN DIEGO
LA JOLLA, CA 92093

COOK, ROBERT L, MPH, MD
PROFESSOR
DEPARTMENT OF EPIDEMIOLOGY
UNIVERSITY OF FLORIDA
GAINESVILLE, FL 32610

CORSI, KAREN F, SCD, MPH
ASSOCIATE PROFESSOR
DEPARTMENT OF PSYCHIATRY
SCHOOL OF MEDICINE
UNIVERSITY OF COLORADO, DENVER
DENVER, CO 80262

CROSBY, RICHARD A, PHD *
ASSOCIATE PROFESSOR
COLLEGE OF PUBLIC HEALTH
UNIVERSITY OF KENTUCKY
LEXINGTON, KY 40506

CUNNINGHAM, CHINAZO , MD
ASSOCIATE PROFESSOR
DIVISION OF GENERAL INTERNAL MEDICINE
MONTEFIORE MEDICAL CENTER
ALBERT EINSTEIN COLLEGE OF MEDICINE
BRONX, NY 10467

EATON, LISA A, PHD *
ASSISTANT PROFESSOR
DEPARTMENT OF PSYCHOLOGY
UNIVERSITY OF CONNECTICUT
STORRS, CT 06029

FUJIMOTO, KAYO , PHD *
ASSISTANT PROFESSOR
HEALTH SCIENCE CENTER
UNIVERSITY OF TEXAS, HOUSTON
HOUSTON, TX 77030

GIORDANO, THOMAS P, MPH, MD
ASSOCIATE PROFESSOR
DEPARTMENT OF MEDICINE
BAYLOR COLLEGE OF MEDICINE
HOUSTON, TX 77030

GWADZ, MARYA , PHD
SENIOR RESEARCH SCIENTIST
COLLEGE OF NURSING
NEW YORK UNIVERSITY
NEW YORK, NY 10003

HIRSHFIELD, SABINA , PHD *
SENIOR RESEARCH SCIENTIST
PUBLIC HEALTH SOLUTIONS
NEW YORK, NY 10013

IANTAFFI, ALEX , PHD *
ASSISTANT PROFESSOR
FAMILY MEDICINE AND COMMUNITY HEALTH
UNIVERSITY OF MINNESOTA
MINNEAPOLIS, MN 55454

KERR, THOMAS , PHD
ASSOCIATE PROFESSOR
DEPARTMENT OF MEDICINE
BRITISH COLUMBIA CENTRE
FOR EXCELLENCE IN HIV/AIDS
UNIVERSITY OF BRITISH COLUMBIA
VANCOUVER, BC V6Z, 1Y6
CANADA

LI, XIAOMING , PHD
PROFESSOR
DEPARTMENT OF PEDIATRICS
SCHOOL OF MEDICINE
WAYNE STATE UNIVERSITY
DETROIT, MI 48201

MERCHANT, ROLAND C, SCD, MPH, MD
ASSOCIATE PROFESSOR
DEPARTMENT OF EMERGENCY MEDICINE
WARREN ALPERT MEDICAL SCHOOL
BROWN UNIVERSITY
PROVIDENCE, RI 02903

MIMIAGA, MATTHEW JAMES, SCD, MPH
ASSOCIATE PROFESSOR
DEPARTMENT OF EPIDEMIOLOGY
SCHOOL OF PUBLIC HEALTH
HARVARD UNIVERSITY
BOSTON, MA 02115

MUSTANSKI, BRIAN , PHD
ASSOCIATE PROFESSOR
DEPARTMENT OF MEDICAL SOCIAL SCIENCES
NORTHWESTERN UNIVERSITY
CHICAGO, IL 60622

NYAMATHI, ADELINE M, PHD
DISTINGUISHED PROFESSOR
SCHOOL OF NURSING
UNIVERSITY OF CALIFORNIA, LOS ANGELES
LOS ANGELES, CA 90095

O'CAMPO, PATRICIA J, PHD *
PROFESSOR AND DIRECTOR
CENTRE FOR RESEARCH ON
INNER CITY HEALTH
ST MICHAEL'S HOSPITAL
TORONTO, ONTARIO, M5B 1W8
CANADA

OTTO-SALAJ, LAURA L, PHD
ASSOCIATE PROFESSOR
DEPARTMENT OF SOCIAL WORK
CENTER FOR ADDICTION, BEHAVIORAL HEALTH RESEARCH
HELEN BADER SCHOOL OF SOCIAL WELFARE
UNIVERSITY OF WISCONSIN, MILWAUKEE
MILWAUKEE, WI 53201

OWNBY, RAYMOND L, MD, PHD
PROFESSOR AND CHAIR
DEPARTMENT OF PSYCHIATRY AND BEHAVIORAL MEDICINE
COLLEGE OF OSTEOPATHIC MEDICINE
NOVA SOUTHEASTERN UNIVERSITY
FORT LAUDERDALE, FL 33314

SORENSEN, JAMES L, PHD *
PROFESSOR
DEPARTMENT OF PSYCHIATRY
SAN FRANCISCO GENERAL HOSPITAL
UNIVERSITY OF CALIFORNIA, SAN FRANCISCO
SAN FRANCISCO, CA 94110

ST LAWRENCE, JANET S, PHD *
PROFESSOR
DEPARTMENT OF PSYCHOLOGY
MISSISSIPPI STATE UNIVERSITY, MERIDIAN
MERIDIAN, MS 39307

SWEAT, MICHAEL D, PHD *
PROFESSOR
DEPARTMENT OF PSYCHIATRY
AND BEHAVIORAL SCIENCES
MEDICAL UNIVERSITY OF SOUTH CAROLINA
CHARLESTON, SC 29426

TURAN, JANET M, PHD *
ASSOCIATE PROFESSOR
DEPARTMENT OF HEALTH CARE ORGANIZATION AND POLICY
SCHOOL OF PUBLIC HEALTH
UNIVERSITY OF ALABAMA AT BIRMINGHAM
BIRMINGHAM, AL 35294

VASSILEVA, JASMIN L, PHD *
ASSOCIATE PROFESSOR
DEPARTMENT OF PSYCHIATRY
INSTITUTE FOR DRUG AND ALCOHOL STUDIES
VIRGINIA COMMONWEALTH UNIVERSITY
RICHMOND, VA 23219

WALDROP-VALVERDE, DRENNA , PHD
ASSOCIATE PROFESSOR
NELL HODGSON WOODRUFF SCHOOL OF NURSING
EMORY UNIVERSITY
ATLANTA, GA 30322

MAIL REVIEWER(S)

HARRIGAN, PAUL RICHARD, PHD
DIRECTOR
DEPARTMENT OF HIV/AIDS
UNIVERSITY OF BRITISH COLUMBIA
VANCOUVER, BC v6t 1z4
CANADA

PATTERSON, THOMAS L, PHD
PROFESSOR
DEPARTMENT OF PSYCHIATRY
UNIVERSITY OF CALIFORNIA, SAN DIEGO
LA JOLLA, CA 92093

SCIENTIFIC REVIEW OFFICER

RUBERT, MARK P, PHD
SCIENTIFIC REVIEW OFFICER
CENTER FOR SCIENTIFIC REVIEW
NATIONAL INSTITUTES OF HEALTH
BETHESDA, MD 20892

EXTRAMURAL SUPPORT ASSISTANT

BOGLEY, DAVID M
LEAD EXTRAMURAL SUPPORT ASSISTANT
CENTER FOR SCIENTIFIC REVIEW
NATIONAL INSTITUTES OF HEALTH
BETHESDA, MD 20892

* Temporary Member. For grant applications, temporary members may participate in the entire meeting or may review only selected applications as needed.

Consultants are required to absent themselves from the room during the review of any application if their presence would constitute or appear to constitute a conflict of interest.
